# Coronavirus disease 2019 (COVID-19) pandemic, central-line–associated bloodstream infection (CLABSI), and catheter-associated urinary tract infection (CAUTI): The urgent need to refocus on hardwiring prevention efforts

**DOI:** 10.1017/ice.2021.70

**Published:** 2021-02-19

**Authors:** Mohamad G. Fakih, Angelo Bufalino, Lisa Sturm, Ren-Huai Huang, Allison Ottenbacher, Karl Saake, Angela Winegar, Richard Fogel, Joseph Cacchione

**Affiliations:** 1 Clinical & Network Services, Ascension Healthcare, St Louis, Missouri; 2 Wayne State University School of Medicine, Detroit, Michigan; 3 Ascension Data Science Institute, Ascension Healthcare, St Louis, Missouri

## Abstract

**Background:**

The coronavirus disease 2019 (COVID-19) pandemic has had a considerable impact on US hospitalizations, affecting processes and patient population.

**Objective:**

To evaluate the impact of COVID-19 pandemic on central-line–associated bloodstream infections (CLABSIs) and catheter associated urinary tract infections (CAUTIs) in hospitals.

**Methods:**

We performed a retrospective study of CLABSIs and CAUTIs in 78 US 12 months before COVID-19 and 6 months during COVID-19 pandemic.

**Results:**

During the 2 study periods, there were 795,022 central-line days and 817,267 urinary catheter days. Compared to the period before the COVID-19 pandemic, CLABSI rates increased by 51.0% during the pandemic period from 0.56 to 0.85 per 1,000 line days (*P* < .001) and by 62.9% from 1.00 to 1.64 per 10,000 patient days (*P* < .001). Hospitals with monthly COVID-19 patients representing >10% of admissions had a National Health Safety Network (NHSN) device standardized infection ratio for CLABSI that was 2.38 times higher than hospitals with <5% prevalence during the pandemic period (*P* = .004). Coagulase-negative *Staphylococcus* CLABSIs increased by 130% from 0.07 to 0.17 events per 1,000 line days (*P* < .001), and *Candida* spp by 56.9% from 0.14 to 0.21 per 1,000 line days (*P* = .01). In contrast, no significant changes were identified for CAUTI (0.86 vs 0.77 per 1,000 catheter days; *P* = .19).

**Conclusions:**

The COVID-19 pandemic was associated with substantial increases in CLABSIs but not CAUTIs. Our findings underscore the importance of hardwiring processes for optimal line care and regular feedback on performance to maintain a safe environment.

The coronavirus disease 2019 (COVID-19) pandemic has had a considerable impact on US healthcare, straining hospital resources and operations. By early December 2020, COVID-19 hospitalizations accounted for ∼14.5% of occupied inpatient beds.^1^ Caring for COVID-19 patients requires more complex processes from diagnostic to safety measures, and it places tremendous pressure on healthcare workers, from staffing challenges^2^ to risk of exposure and infection. In addition, the inpatient population has changed,^3^ with a precipitous drop in elective surgical cases^4^ and an avoidance of patients being admitted for other medical conditions resulting in a higher case mix index (CMI) among our patient populations. Furthermore, COVID-19 patients often required close monitoring and higher levels of care, including ventilatory and critical care support.^5^

Hospital-onset healthcare-associated infections (HAIs) are key quality and safety metrics publicly reported in the acute-care space and linked to hospital reimbursement by the Centers for Medicare and Medicaid Services (CMS).^6^ National efforts have been successfully implemented to reduce central-line–associated bloodstream infection (CLABSI)^7^ and catheter-associated urinary tract infection (CAUTI).^8^ We evaluated the impact of COVID-19 pandemic on the Centers for Disease Control and Prevention (CDC) National Healthcare Safety Network (NHSN) CLABSI and CAUTI outcomes in 78 hospitals of a large, multistate, healthcare system in the United States. Particularly, we examined changes in device utilization, CLABSI and CAUTI events, the associated microbiology of these infections, and the impact on outcomes reflected by the NHSN standardized infection ratio.

## Methods

### Setting

This study was a retrospective evaluation of CLABSI and CAUTI outcomes in 78 hospitals from a single healthcare system over 2 periods: before the COVID-19 pandemic (March 2019–February 2020; 12 months) and during the COVID-19 pandemic (March–August 2020; 6 months). We evaluated whether increases in CLABSI or CAUTI events occurred with the COVID-19 pandemic and whether the microbiology of the associated organisms changed.

### Relation between device-associated events and the COVID-19 pandemic

To compare CLABSI and CAUTI outcomes before the COVID-19 pandemic with those during the COVID-19 pandemic period, we evaluated CLABSI and CAUTI events as well as central-line and urinary catheter utilization reflected by their standardized utilization ratios (SURs). We also examined the prevalence of COVID-19 admissions per month and its relationship to CLABSI and CAUTI events. Hospitals were classified based on the number of beds as large (>300), medium (100–300), or small (<100). For each month during the pandemic period, hospitals were categorized as having low (≤5% of hospital admits), mid-level (>5% to 10% of hospital admits), or high (>10% of hospital admits) COVID-19 prevalence. The device standardized infection ratio (dSIR) and the population SIR (pSIR) were compared for the 2 periods.^9^ The pSIR accounts for changes in device use and its effect on infection events for a population. The pSIR is the ratio of observed events divided by predicted events based on the predicted device days for the same population. At the unit level, it may be calculated by multiplying the dSIR by the SUR.

### Microbiology

We reviewed the associated organisms (up to 3 per CLABSI event and up to 2 per CAUTI event) documented in the NHSN database before and during the pandemic. Specifically, we looked at differences in proportions of gram-positive bacteria, gram-negative bacteria, and yeast over the 2 periods. We also compared the CLABSI rates of specific organisms before and during the pandemic.

### Population

We included hospitalized patients who had a central-line or a urinary catheter during their stay between March 2019 and August 2020 reporting to the NHSN from 78 hospitals. All patients with a CDC NHSN–defined CLABSI or CAUTI event between March 2019 and August 2020 were identified. The NHSN data included patients with events (CLABSIs and CAUTIs), device and patient days over period of study, the type of unit where the events occurred (ie, intensive care vs non–intensive care), and associated microorganisms.

### Data sources

The CDC NHSN database was used to identify patients with CLABSIs and/or CAUTIs. The Ascension COVID-19 patient data set was used to identify patients admitted with COVID-19, including sociodemographic characteristics, laboratory results, and healthcare utilization as captured in electronic health records and administrative data. The final data were deidentified and evaluated as aggregates. The study underwent institutional review board evaluation and was deemed to be exempt from further review.

### Statistical analysis

We used χ^2^ tests to evaluate the associations of the COVID-19 pandemic with CLABSI and CAUTI events. Statistical significance was assessed as *P* < .05, and analyses were performed using R version 3.6.2 software (R Foundation for Statistical Computing, Vienna, Austria). These analyses were further stratified by events occurring within or outside intensive care and by hospital size. Line utilization, predicted utilization, reported infections, and predicted infections were calculated for SUR, dSIR, and pSIR. CLABSI dSIR, and CAUTI dSIR were calculated and compared based on monthly levels of COVID-19 admissions. The event rates associated with organisms based on device days were compared for periods before versus during the COVID-19 pandemic.

## Results

We compared data from the 12 months before (March 2019–February 2020) and 6 months during (March–August 2020) COVID-19 pandemic in 78 hospitals in 12 states: <100 beds (n = 21); 100–300 beds (n = 29); and >300 beds (n = 28). Overall, the study included 795,022 central-line days (utilization, 18.3%) and 817,267 urinary catheter days (utilization, 18.4%). The SURs over the full 18 months of the study were 0.89 for central lines and 0.80 for urinary catheters, and the dSIRs were 0.68 for CLABSI and 0.69 for CAUTI.

### Comparing device infection over the 2 periods

Compared to the pre–COVID-19 period, CLABSI rates increased by 51.0% during the pandemic period from 0.56 to 0.85 per 1,000 line days (*P* < .001) and by 62.9% from 1.00 to 1.64 per 10,000 patient days (*P* < .001) (Table 1). This trend was mainly observed in the intensive care units (ICUs) where CLABSI rates increased by 71.0% from 0.68 to 1.16 per 1,000 line days (*P* < .001) and by 90.7% from 2.95 to 5.63 per 10,000 patient days (*P* < .001). Larger hospitals (>300 beds) were most affected, with 154 events during the pandemic period, increasing from 0.58 to 0.88 per 1,000 line days (*P* < .001) and from 1.07 to 1.74 per 10,000 patient days (*P* < .001). Medium-sized hospitals had similar increases in CLABSIs from 0.54 to 0.82 per 1,000 line days (*P* = .009) and from 0.95 to 1.61 per 10,000 patient days (*P* = .001). No significant changes were detected in small hospitals.

**Table 1.** Central-Line–Associated Bloodstream Infection (CLABSI) and Catheter-Associated Urinary Tract Infection (CAUTI) Rates Before and During the COVID-19 Pandemic

Variable	Pre–COVID-19 (Mar 2019–Feb 2020)					COVID-19 (Mar–Aug 2020)					Pre–COVID-19 vs COVID-19 Relative Change, % (*P* Value)	
	Observed Events	Device Days	Rate per 1,000 Device Days	Patient Days	Rate per 10,000 Patient Days	Observed Events	Device Days	Rate per 1,000 Device Days	Patient Days	Rate per 10,000 Patient Days	Rate per 1,000 Device Days	Rate per 10,000 Patient Days
**CLABSI**	**302**	**537,124**	**0.56**	**3,007,609**	**1.00**	**219**	**257,898**	**0.85**	**1,338,683**	**1.64**	**51.0 (<.001)**	**62.9 (<.001)**
Intensive care	149	220,491	0.68	504,344	2.95	141	122,009	1.16	250,268	5.63	71.0 (<.001)	90.7 (<.001)
Non–intensive care	153	316,633	0.48	2,503,265	0.61	78	135,889	0.57	1,088,415	0.72	18.8 (.22)	17.3 (.25)
**CAUTI**	**471**	**546,952**	**0.86**	**3,070,946**	**1.53**	**209**	**270,315**	**0.77**	**1,366,558**	**1.53**	**−10.2 (.19)**	**−0.3 (.97)**
Intensive care	213	242,101	0.88	504,344	4.22	115	128,234	0.90	250,268	4.60	1.9 (.87)	8.8 (.47)
Non–intensive care	258	304,851	0.85	2,566,602	1.01	94	142,081	0.66	1,116,290	0.84	−21.8 (.04)	−16.2 (.14)

In contrast, overall CAUTI rates did not show any significant difference between the pre–COVID-19 (0.86 per 1,000 catheter days) and COVID-19 periods (0.77 per 1,000 catheter days; *P* = .19). We detected no significant differences within the ICUs, but we detected an improvement in CAUTI rates in the non-ICU setting from the pre–COVID-19 period normalized to catheter days (0.85 per 1,000 catheter days) to the pandemic period (0.66 per 1,000 catheter days; *P* = .04). We detected no associated differences in CAUTI rates based on patient days in the non-ICUs.

### Evaluating the changes in SUR and its effect on dSIR and pSIR

Both central-line and urinary catheter SURs increased during the pandemic period. There was a 4.9% increase in central-line SUR from 0.88 before the COVID-19 pandemic to 0.92 during the pandemic (*P* < .001). Urinary catheter SUR increased by 7.4%, from 0.79 before COVID-19 to 0.84 during the pandemic (*P* < .001) (Table 2). The dSIR for CLABSI increased 49.6% during the pandemic, from 0.58 before COVID-19 to 0.87 during the pandemic (*P* < .001). This change was more pronounced when we calculated the pSIR, which increased 58.4% from 0.51 before the pandemic to 0.81 during the pandemic (*P* < .001). For CAUTI, we detected no significant changes in dSIR; the pre–COVID-19 dSIR was 0.71 and the dSIR during the pandemic was 0.64 (*P* = .21). Nor did we detect a significant change in the pSIR for CAUTI; the pre–COVID-19 pSIR was 0.58 and the pSIR during the pandemic was 0.57 (*P* = .73).

**Table 2.** SURs and SIRs for CLABSIs and CAUTIs Before and During the COVID-19 Pandemic

Variable	Pre–COVID-19 (Mar 2019–Feb 2020)			COVID-19 (Mar–Aug 2020)			Pre–COVID-19 vs COVID-19
SUR	Utilization	Predicted Utilization	Ratio	Utilization	Predicted Utilization	Ratio	Relative Change, % (*P* Value)
Central-line SUR	537,124	609,722	0.88	257,898	279,143	0.92	4.9 (<.001)
Urinary catheter SUR	546,952	695,362	0.79	270,315	319,929	0.84	7.4% (<.001)
SIR	Reported Infections	Predicted Infections	Ratio	Reported Infections	Predicted Infections	Ratio	Relative Change, % (*P* Value)
CLABSI dSIR^a^	302	520	0.58	219	252	0.87	49.6 (<.001)
CLABSI pSIR^b^	302	593	0.51	219	272	0.81	58.4 (<.001)
CAUTI dSIR^a^	471	660	0.71	209	325	0.64	−9.8 (.21)
CAUTI pSIR^b^	471	807	0.58	209	368	0.57	−2.8 (.73)

Note. SUR, standardized utilization ratio; SIR, standardized infection ratio; CLABSI, central-line–associated bloodstream infection; CAUTI, catheter-associated urinary tract infection;

a Device standardized infection ratio.

b Population standardized infection ratio.

### Correlations between COVID-19 hospital prevalence, CLABSI, and CAUTI events

During months when patients with active COVID-19 represented >10% of admissions, the CLABSI dSIR was 2.38 times higher (dSIR, 1.58) than in months when COVID-19 prevalence among hospitalized patients was <5% (dSIR, 0.67; *P* = .004) (Table 3). We detected no significant differences in CAUTI dSIR based on monthly COVID-19 prevalence.

**Table 3.** CLABSI and CAUTI Device SIRs and COVID-19 as a Proportion of Admissions During Pandemic Period

Monthly COVID-19 Prevalence^a^	CLABSI dSIR	*P* Value	CAUTI dSIR	*P* Value
High (>10% of hospital admissions)	1.58	Reference	0.61	Reference
Mid (>5%–10% of hospital admissions)	1.09	.05	0.70	.55
Low (≤5% of hospital admissions)	0.67	.004	0.64	.64

Note. CLABSI, central-line–associated bloodstream infection; CAUTI, catheter-associated urinary tract infection; SIR, standardized infection ratio.

a Patient-based COVID-19 prevalence was available for 76 of 78 hospitals.

### CLABSI events and outcomes during the COVID-19 period

In total, 219 CLABSI events occurred between March and August 2020; of these, 52 (24%) occurred in patients hospitalized with COVID-19. Also, 2 additional patients were identified with COVID-19 after their CLABSI event. The average time to CLABSI from COVID-19 diagnosis was 18.0 days (median, 15.0). During the 6 months evaluated during the pandemic, 18,048 patients diagnosed with COVID-19 were admitted to the 76 of the 78 hospitals (with available data on patient-based COVID-19 prevalence), constituting 5.1% of all admissions. Proportionately, COVID-19 patients had >5 times more CLABSI events than non–COVID-19 patients. A significant difference was observed in mortality between patients with CLABSI for those with COVID-19 (28 of 52, 53.8%) and without COVID-19 (40 of 167, 24.0%) during that period (*P* < .001).

### Microbiology associated with CLABSIs and CAUTIs

Overall, 344 organisms were associated with CLABSI events and 535 for CAUTI events in the 12 months before the COVID-19 pandemic, while 236 organisms were associated with CLABSI events and 237 organisms were associated with CAUTI events during the 6 months during the COVID-19 pandemic (Table 4). Gram-positive CLABSIs increased by 80.6% from 0.27 to 0.48 events per 1,000 line days (*P* < .001). Specifically, coagulase-negative *Staphylococcus* CLABSIs increased by 130% from 0.07 to 0.17 events per 1,000 line days (*P* < .001), and *Candida* spp infections increased by 56.9% from 0.14 to 0.21 per 1,000 line days (*P* = .01). We did not observe any significant changes between the 2 periods for organisms associated with CAUTIs.

**Table 4.** Comparing Events Based on Organisms for CLABSI and CAUTI Pre–COVID-19 to Pandemic Periods

Variable	Before COVID-19 (March 2019–February 2020)			During the COVID-19 Pandemic (March-August 2020)			Before COVID-19 vs During COVID-19	
	No. of Events (Organisms^a^)	Device Days	Rate (Events per 1,000 Device Days)	No. of Events (Organisms^a^)	Device Days	Rate (Events per 1,000 Device Days)	*P* Value	Relative Change, %
**CLABSI**	**302 (344)**	**537,124**	**0.56**	**219 (236)**	**257,898**	**0.85**	**<.001**	**51.0**
Gram-positive organisms	143 (155)	537,124	0.27	124 (129)	257,898	0.48	<.001	80.6
* Staphylococcus aureus*	53 (53)	537,124	0.10	33 (33)	257,898	0.13	.24	29.7
Methicillin resistant *Staphylococcus aureus*	21 (21)	537,124	0.04	16 (16)	257,898	0.06	.16	58.7
* *Coagulase-negative *Staphylococcus*	39 (42)	537,124	0.07	43 (44)	257,898	0.17	<.001	129.6
Gram-negative organisms	103 (112)	537,124	0.19	48 (50)	257,898	0.19	.86	−2.9
*Candida* spp	73 (76)	537,124	0.14	55 (55)	257,898	0.21	.01	56.9
**CAUTI**	**471 (535)**	**546,952**	**0.86**	**209 (237)**	**270,315**	**0.77**	**.19**	−**10.2**
Gram-positive organisms	100 (106)	546,952	0.18	52 (52)	270,315	0.19	.77	5.2
Gram-negative organisms	392 (429)	546,952	0.72	168 (185)	270,315	0.62	.12	−13.3

Note. CLABSI, central-line–associated bloodstream infection; CAUTI, catheter-associated urinary tract infection.

a Rates are based on events; >1 organism may be associated with an event.

## Discussion

The COVID-19 pandemic has had a disruptive effect on the US healthcare system, resulting in an abrupt drop in admissions for most common conditions and leading to a selective increase in severity of illness among hospitalized patients.^3^ As a large US health system with 78 hospitals, we report our experience and the outcomes related to 2 devices: central lines and urinary catheters. Our data show differing impacts of COVID-19 on the CDC NHSN–defined rates of CLABSI and CAUTI events. Based on central-line days, CLABSI rates increased by more than two-thirds within the ICUs, whereas the events per 10,000 patient days almost doubled. Recent studies have reported large increases in CLABSI events^10^ and a higher likelihood for secondary bloodstream infections in COVID-19 patients requiring intensive care.^11^ In an attempt to reduce healthcare worker exposure to patients and to preserve personal protective equipment, the frequency of contact with patients may have changed during the pandemic. Moreover, patients admitted during the pandemic were more likely to require critical care support and to need it for a longer period of time,^12^ potentially putting them at greater risk for CLABSI. The proportion of COVID-19 patients with CLABSI events was 5 times greater than for non–COVID-19 patients during the pandemic period. Additionally, the average time from COVID-19 diagnosis to developing CLABSI was ∼18 days, indicating that the CLABSI events occurred in COVID-19 patients with prolonged hospitalization. Although the increases in CLABSI events did not reach statistical significance in non–ICUs, we expect that similar challenges in infection prevention were encountered.

An integral risk-reduction strategy for CLABSI is anchored in the optimal maintenance of the device.^13^ In the prepandemic period, CLABSI prevention strategies were hardwired at our hospitals leading to a dSIR of 0.58, which outperformed the national CDC NHSN CLABSI dSIR of 0.69 for 2019.^14^ The pandemic likely affected both the care of the line for COVID-19 and non–COVID-19 hospitalized patients. Qualitative feedback from infection prevention teams reported changes to routine CLABSI prevention practices in ICUs, such as less universal decolonization (eg, mupirocin administration and chlorhexidine bathing), alterations in line care due to intravenous pumps placed in hallways (eg, extension tubing used and less bedside checks on lines), line and dressing integrity gaps related to prone positioning of patients, opportunities in scrub-the-hub compliance, and increases in line draws for blood cultures. Another variable potentially impacting CLABSI outcomes includes staffing changes responding to increased patient volume on the units, such as the help of traveling clinicians not as familiar with standard unit prevention practices. Lastly, during the pre–COVID-19 period, “line rounds” were a routine practice, ensuring that proper device selection, utilization, and bedside practices were being followed. The teams reported that many of those rounds stopped during the COVID-19 pandemic period due to competing priorities. On the other hand, with the less intense needs for indwelling urinary catheter device care, nominal changes in practice were reported, with the exception of some reports of increases in pan-culturing for febrile patients.

In addition to the increases in the occurrence of CLABSI during the pandemic, we observed changes in the microbiology associated with these events. Gram-positive–associated CLABSIs increased by >80% during the pandemic period, and coagulase-negative *Staphylococcus*–associated CLABSIs more than doubled. This change may indicate the increased risk for line infection and contamination due to suboptimal aseptic practices while obtaining blood cultures in a stressful environment. The CDC NHSN definition of CLABSI^15^ includes common commensal organisms identified in 2 or more blood cultures in the presence of at least fever, chills, and/or hypotension, and no other related site of infection. The classification may overestimate CLABSI in the setting of a high contamination rate in a severely ill population. A higher incidence of blood culture contamination with commensal organisms, particularly coagulase negative staphylococci, has been reported in hospitalized patients with COVID-19.^16^ Another noteworthy observation was the increase in *Candida* spp–associated CLABSIs. Broad-spectrum antimicrobial pressure and prolonged use of central lines may contribute to higher candidemia risk.^17^ We did not find increases in gram-negative CLABSIs.

Our CAUTI findings differed from those for CLABSI. During the pandemic, we did not witness an increase in CAUTI events. The CDC NHSN definition of CAUTI^15^ includes the identification of no more than 2 organisms with a quantitative culture of >100,000 colony-forming units per milliliter, in the presence of focal findings or fever. In addition, it excludes candiduria from the definition. The NHSN CAUTI definition is more dependent on culturing practices and the preexisting prevalence of bacteriuria and is less susceptible to device maintenance.^18-20^ Plausible explanations for these findings include different hospitalized patient populations (more COVID-19; fewer surgical and elective admissions), increases in antimicrobial use resulting in the suppression of bacteriuria, and a reduction in inappropriate triggers to ordering urine cultures (eg, urine color or turbidity)^21^ in an effort to minimize contact between healthcare workers and patients.

Finally, we used the risk-adjusted dSIR and pSIR to evaluate the changes in CLABSIs and CAUTIs between the 2 periods. In the pandemic period, we detected a significant increase in device use, reflected by the SUR, for both central lines and urinary catheters. These findings potentially indicate a sicker patient population. In addition, the dSIRs and pSIRs showed commensurate increases in CLABSI events and infection rates normalized to line days and patient days. Traditionally, the dSIR has been used to evaluate the risk of the device among patients who are exposed to it, but it fails to capture the risk to the whole hospitalized population. Our findings indicate that the impact on CLABSI during the pandemic period was more pronounced when the pSIR is considered, which better reflects the magnitude of the events accounting for device use.^9^

Our study raises the importance of obtaining a better understanding of the susceptibility of NHSN reported outcome measures to changes in practices or population served. Due to the pandemic, the CMS allowed voluntary reporting and submission of NHSN HAI measure data through the Extraordinary Circumstances Exceptions.^22^ We made the decision as a system to not interrupt submission of NHSN data from our participating sites. Our continued reporting allowed us to identify the impact of the COVID-19 pandemic on HAIs, giving us the opportunity to present 2 different tales for the impact of the COVID-19 pandemic on outcomes associated with central-line and urinary catheter devices. The Interim Final Rule in August 2020 clarified that CMS will not use data submitted from January through June 2020 to calculate performance in its hospital quality reporting penalty and incentive programs.^23^ Future work is needed to better delineate each of these surveillance metrics as surrogates to final clinical outcomes used for ongoing public reporting and hospital-acquired conditions.^6^ However, we believe that public reporting, while exempting hospitals from penalties, is an important advocacy and policy consideration for future pandemics or unforeseeable events that may affect hospital quality outcomes.

Our study has some limitations. Particularly for those facilities that experienced surges in COVID-19 admissions, it became necessary per NHSN guidance to revise the mapping of certain locations within the NHSN database based on patient population. Despite the technical challenges with location mapping, we do not believe that this factor had an impact on our overall findings. We have observed the increases in CLABSI events occur predominantly in the ICUs. Moreover, the overall increases in CLABSI were accounted for when evaluating by rates and SIRs. Also, we studied the experience of 1 health system with a history of focus on reducing healthcare-associated infections. Prior to the COVID-19 pandemic, we had implemented systemwide initiatives to reduce CLABSI risk, from standardizing policies of insertion and maintenance, to optimizing placement and maintenance kits and hardwiring competencies. The gaps and events may be even more pronounced in other acute-care facilities. In addition, other health systems and hospitals may have chosen not to report during the pandemic, which may lead to the underestimation of the impact of the pandemic on CLABSIs nationally. On the other hand, our study represents the largest evaluation of device infections during the COVID-19 pandemic in the United States to date. The large number of hospitals included, and their diverse geographic locations and patient populations, makes it reasonable to generalize our findings.

We conclude that the COVID-19 pandemic has had a significant impact on CLABSIs but not on CAUTIs within our system. Our results underscore the importance of ensuring close monitoring of processes and outcomes related to device use during the pandemic and providing regular feedback on performance to the frontline staff and clinical leaders to ensure a safe environment to care for all our hospitalized patients. These results also highlight the importance of avoiding any disruption of public reporting of healthcare-associated infections. We must learn from the COVID-19 pandemic and design processes resilient to any unpredictable event.
